# Expanded Newborn Screening for Inborn Errors of Metabolism by Tandem Mass Spectrometry in Suzhou, China: Disease Spectrum, Prevalence, Genetic Characteristics in a Chinese Population

**DOI:** 10.3389/fgene.2019.01052

**Published:** 2019-10-29

**Authors:** Ting Wang, Jun Ma, Qin Zhang, Ang Gao, Qi Wang, Hong Li, Jingjing Xiang, Benjing Wang

**Affiliations:** ^1^Newborn Screening Laboratory, Center for Reproduction and Genetics, the Affiliated Suzhou Hospital of Nanjing Medical University, Suzhou, China; ^2^Genetic Clinic, Center for Reproduction and Genetics, the Affiliated Suzhou Hospital of Nanjing Medical University, Suzhou, China; ^3^Infertility Clinic, Center for Reproduction and Genetics, the Affiliated Suzhou Hospital of Nanjing Medical University, Suzhou, China; ^4^Genetic Laboratory, Center for Reproduction and Genetics, the Affiliated Suzhou Hospital of Nanjing Medical University, Suzhou, China

**Keywords:** expanded newborn screening, inborn errors of metabolism, tandem mass spectrometry, disease spectrum, prevalence, genetic characteristics, hotspot mutation

## Abstract

Expanded newborn screening for inborn errors of metabolism (IEMs) by tandem mass spectrometry (MS/MS) could simultaneously analyze more than 40 metabolites and identify about 50 kinds of IEMs. Next generation sequencing (NGS) targeting hundreds of IMEs-associated genes as a follow-up test in expanded newborn screening has been used for genetic analysis of patients. The spectrum, prevalence, and genetic characteristic of IEMs vary dramatically in different populations. To determine the spectrum, prevalence, and gene mutations of IEMs in newborns in Suzhou, China, 401,660 newborns were screened by MS/MS and 138 patients were referred to genetic analysis by NGS. The spectrum of 22 IEMs were observed in Suzhou population of newborns, and the overall incidence (excluding short chain acyl-CoA dehydrogenase deficiency (SCADD) and 3-Methylcrotonyl-CoA carboxylase deficiency (3-MCCD)) was 1/3,163. The prevalence of each IEM ranged from 1/401,660 to 1/19,128, while phenylketonuria (PKU) (1/19,128) and Mild hyperphenylalaninemia (M-HPA) (1/19,128) were the most common IEMs, followed by primary carnitine uptake defect (PCUD) (1/26,777), SCADD (1/28,690), hypermethioninemia (H-MET) (1/30,893), 3-MCCD (1/33,412) and methylmalonic acidemia (MMA) (1/40,166). Moreover, 89 reported mutations and 51 novel mutations in 25 IMEs-associated genes were detected in 138 patients with one of 22 IEMs. Some hotspot mutations were observed for ten IEMs, including *PAH* gene c.728G > A, c.611A > G, and c.721C > T for Phenylketonuria, *PAH* gene c.158G > A, c.1238G > C, c.728G > A, and c.1315+6T > A for M-HPA, *SLC22A5* gene c.1400C > G, c.51C > G, and c.760C > T for PCUD, *ACADS* gene c.1031A > G, c.164C > T, and c.1130C > T for SCAD deficiency, *MAT1A* gene c.791G > A for H-MET, *MCCC1* gene c.639+2T > A and c.863A > G for 3-MCCD, *MMUT* gene c.1663G > A for MMA, *SLC25A13* gene c.IVS16ins3Kb and c.852_855delTATG for cittrullinemia II, *PTS* gene c.259C > T and c.166G > A for Tetrahydrobiopterin deficiency, and *ACAD8* gene c.1000C > T and c.286C > A for Isobutyryl coa dehydrogenase deficiency. All these hotspot mutations were reported to be pathogenic or likely pathogenic, except a novel mutation of *ACAD8* gene c.286C > A. These mutational hotspots could be potential candidates for gene screening and these novel mutations expanded the mutational spectrum of IEMs. Therefore, our findings could be of value for genetic counseling and genetic diagnosis of IEMs.

## Introduction

Inborn errors of metabolism (IMEs) are a large group of monogenic diseases resulting in death and abnormalities of physical and neurological development at almost all stages of life. IMEs are always caused by the defect of an enzyme, its coenzyme, or a transporter leading to the accumulation of its substrate and/or the insufficiency of its downstream products. Nowadays, the introduction of tandem mass spectrometry (TMS) allows screening for more than 50 IMEs using dried blood spot in the neonatal period (Therrell et al., 2015). For the neonates screened to have IMEs, some serious clinical consequence could be prevented, including mild to severe irreversible mental retardation, lifelong disability, physical handicaps, coma, and early death, if early diagnosis and treatment were implemented (Therrell et al., 2015). Therefore, expanded newborn screening program covering dozens of diseases has been implemented in the majority of developed countries. For example, population-based data are available in the United States (Centers for Disease Control and Prevention, 2008; Gallant et al., 2012; Hsu et al., 2013), Canada (Karaceper et al., 2016), United Kingdom (Sanderson et al., 2006), Germany (Lindner et al., 2008), France (Dhondt, 2010), Egypt (Hassan et al., 2016), Greece (Loukas et al., 2010), Saudi Arabia (Alfadhel et al., 2017), Australia (Wiley et al., 1999; Webster et al., 2003), South Korea (Yoon et al., 2005), Singapore (Lim et al., 2014), and Japan (Shibata et al., 2018).

The advent of target capture and next generation sequencing (NGS) enables simultaneously sequence a large group of targeted genes accounting for numerous diseases, which has become the best choice for identification of genetic etiology of IMEs following expanded newborn screening program. The utility of NGS in expanded newborn screening has enriched our understanding of genetic etiology, genetic characteristics, and phenotype-genotype correlation of IMEs. Some hotspot variants resulting in the defect of enzymes have been identified in patients with IMEs, such as *ACADS* variants c.511C > T and c.625G > A for short chain acyl-CoA dehydrogenase deficiency (SCADD; MIM# 201470) (Tonin et al., 2016; Nochi et al., 2017), *PAH* variant c.728C > A for phenylketonuria (PKU; MIM# 261600) (Liu et al., 2017), and so on. Also, many IMEs have a dramatic variation of symptoms and the outcome of the affected patients was correlated with genotype, such as medium chain acyl-CoA dehydrogenase deficiency (MCADD; MIM# 201450) (Ensenauer et al., 2005; Maier et al., 2005), very long chain acyl-CoA dehydrogenase deficiency (VLCADD; MIM# 609016) (Andresen et al., 1999; Obaid et al., 2018), and so on. In addition, the spectrum, the incidence, and the genetic characteristics of IMEs vary dramatically in different regions and populations.

Expanded newborn screening was introduced in China in 2004, later than developed countries. In the milestone pilot study, a total of 371,942 newborns were screened in four centers, and the collective estimated incidence of overall IMEs was 1/3,795 in live births, with a sensitivity of 98.99% and a specificity of 99.83% (Shi et al., 2012). Recently, targeted sequencing of genes associated to more than 50 IMEs by NGS was used as a follow-up test for genetic diagnosis after the expanded newborn screening, and some novel variants were found in Chinese patients. In Suzhou, the expanded newborn screening program targeting 27 IMEs started in 2014. Until now, its screening rate is closed to 100% of live births and more than 400,000 newborns have been referred to expand newborn screening. A total of 22 kinds of IMEs were identified in Suzhou population and 153 infants were diagnosed with one of these IMEs. Almost all these patients were referred to genetic analysis *via* targeted NGS. 140 variants in 25 IMEs-associated genes were found in 138 patients. Some hotspot variants were also observed in Suzhou patients, including c.791G > A in *MATA1* gene for hypermethioninemia (MIM# 250850), c.158G > A in *PAH* gene for mild hyperphenylalaninemia (M-HPA; MIM# 261600), c.721C > T in *PAH* gene for PKU (MIM# 261600), c.852_855delTATG in *SLC25A13* gene for citrullinemia type II (CTLN 2; MIM# 605814), c.639+2T > A in *MCCC1* gene for 3- methylcroton acyl coenzyme A carboxylase deficiency (3-MCCD; MIM# 210200 and 210210), c.1400C > G in *SLC22A5* gene for primary carnitine uptake defect (PCUD; MIM# 212140), and c1031A > G in *ACADS* gene for SCADD. These hotspot mutations could explain the relative high incidence of associated IMEs. As a result, it is critical to screen these mutations and prenatal genetic consulting for Suzhou population. These mutations are good candidates for further research on genetic characteristics in other Chinese populations.

## Material and Methods

### Subjects

**Figure 1:**
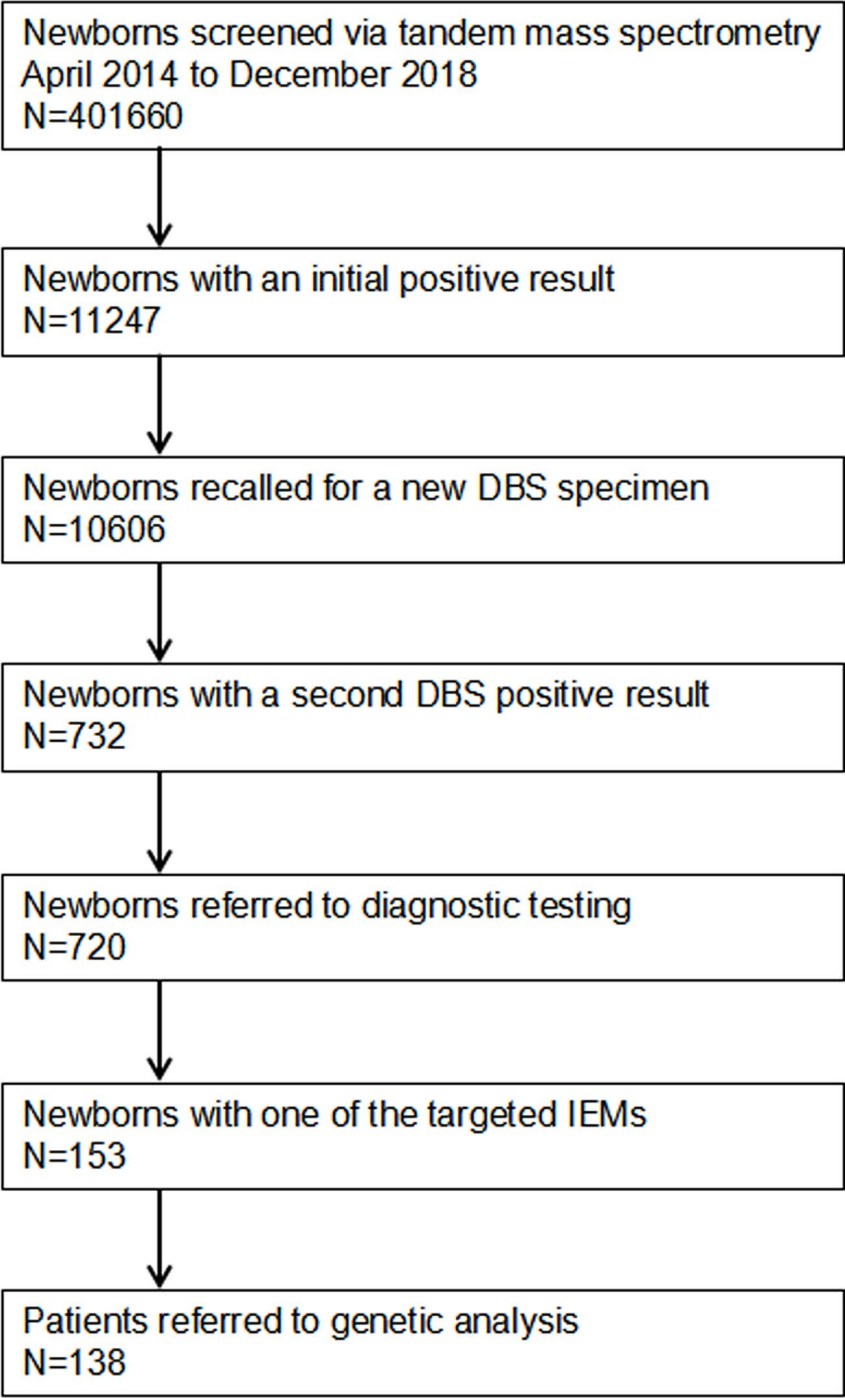
Flowchart of expanded newborn screening for inborn errors of metabolism and genetic analysis of patients. DBS, dried blood spot; IEMs, inborn errors of metabolisms.

A total of 401,660 newborns were referred to expand newborn screening. Informed and written consent was obtained from the parents of all screened newborns. Our screened protocol is consistent with other newborn screening centers in China, and was shown in **Figure 1**. The protocol was reviewed and approved by Ethic committee of the Affiliated Suzhou Hospital of Nanjing Medical University.

### Expanded Newborn Screening Assay

Eleven amino acids, 30 acylcarnitines, free carnitine, and succinylacetone were tested using tandem mass spectrometry (**Supplementary Table 1**). Assays for screening inborn metabolism disorders were performed using screening kit (PerkinElmer, USA) and Waters HPLC-tandem mass spectrometry (TQD, Waters, USA). In brief, 100-ul extract liquor containing internal standards was added into U bottom plates. After incubating for 45 min at 45°C, 75-ul extract liquor was transferred into V bottom plates. After 2 h standing at ambient temperature, 25-ul liquor was injected into tandem mass spectrometry for metabolites analyses. Three levels of internal quality controls including blank, low, and high were used for quality control.

### Positive Results for IMEs

In our screening panel, 26 kinds of IEMs were included. Each IEM had two or more indicators including metabolites and ratios, and their cut-off values. When DBS results met the positive rules of IEMs, they were considered as positive. All the positive rules of IEMs were shown in **Table 1**.

**Table 1:** Conditions and their positive rules in expanded newborn screening panel.

Conditions	Positive rule 1	Positive rule 2	Positive rule 3
PKU, M-HPA, BH4 deficiency	PHE > 100 nmol/L, PHE/TYR > 1.2	PHE > 130 nmol/L,	PHE/TYR > 2
HCY, H-MET	MET > 70 nmol/L	MET > 43 nmol/L, MET/PHE > 0.85	
ASA	CIT > 50 nmol/L	CIT > 35 nmol/L, ALA/CIT < 8.5	
MSUD	LEU+ILE+PRO-OH > 400 nmol/L	LEU+ILE+PRO-OH > 320 nmol/L, LEU+ILE+PRO-OH/PHE > 5.9, VAL > 250 nmol/L	
OTC	CIT < 5.5 nmol/L	CIT < 6.5nmol/L, CIT/PHE < 0.12	
H-ORN	ORN > 450 nmol/L	ORN > 340 nmol/L, ORN/CIT > 24	
H-TYR	TYR > 400 nmol/L	TYR > 350nmol/L, LEU+ILE+PRO-OH/TYR < 0.5, PHE/TYR < 0.15	
H-ARG	ARG > 65 nmol/L	ARG/PHE > 1.2, ARG > 50 nmol/L	
TYR-I	SA > 2 nmol/L	SA > 1.2 nmol/L, SA/PHE > 0.03	
H-PRO	PRO > 470 nmol/L		
MUT, PROP	C3/C0 > 0.3 nmol/L	C3/C2 > 0.21, C3 > 4.5nmol/L	C3 > 6.5
IVA, 2MBG	C5 > 0.8 nmol/L	C5 > 0.4 nmol/L, C5/C0 > 0.02	
3-MCC, MCD, 2M3HBA, 3MGA, HMG	C4DC+C5-OH > 0.7nmol/L	C4DC+C5-OH > 0.5nmol/L, (C4DC+C5-OH)/C0 > 0.025	
MADD	C5 > 0.4 nmol/L,C4 > 0.5 nmol/L		
BKT	C5:1 > 0.02nmol/L,C4DC+C5-OH > 0.5nmol/L		
MAL	C3DC+C4-OH > 0.8nmol/L	C3DC+C4-OH > 0.45nmol/L, (C3DC+C4-OH)/C10 > 5	
GA-I	C5DC+C6-OH > 0.4nmol/L	C5DC+C6-OH > 0.23nmol/L, (C5DC+C6-OH)/(C3DC+C4-OH) > 2, (C5DC+C6-OH)/(C4DC+C5-OH) > 1.38	
MCADD	C8 > 0.3nmol/L	C6 > 0.11nmol/L, C8 > 0.19nmol/L, C8/C2≥0.01, (C4DC+C5-OH)/C8 < 1	
VLCADD	C14:1 > 0.5nmol/L	C14:1 > 0.35nmol/L, C14:1/C16 > 0.14, C14:1/C2≥0.02	
LCHADD, TFP	C16-OH > 0.06nmol/L,C16-OH/C16 > 0.025,C18:1-OH > = 0.06 nmol/L,C18-OH > 0.03 nmol/L		
PCUD	C0 < 9.5 nmol/L		
CPT-Ia	C0 > 100 nmol/L	C0/(C16+C18) > 50, C0 > 55 nmol/L, (C16+C18:1)/C2 < 0.08	
CPY-II, CACT	C18 > 1.9 nmol/L, C18:1 > 3 nmol/L	C16> 12 nmol/L, C16 > 7 nmol/L, C18:1 > 3 nmol/L	
SCADD	C4 > 0.7 nmol/L	C4 > 0.5 nmol/L, C4/C2 > 0.03	
NKHG	GLY > 1100 nmol/L		
IBG, EMA	C4 > 0.7 nmol/L	C4/C3 > 0.45, C4/C2 > 0.03	

### Genetic Analysis

High throughput sequencing was performed on all patients diagnosed with one kind of IEMs using the expanded edition panel of IMEs (Genuine Diagnostic, Hangzhou, China) including 306 genes related to IEMs. In brief, the target sequences were enriched using Agilent SureSelect Human Exon Sequence Capture Kit (Agilent Technologies, Inc, California, USA). Next, the captured products were purified using Agencourt AMPure XP beads (Beckman Coulter, Inc, Miami, USA). Then, the sequencing library was established using TruePrepTM DNA Library Prep Kit V2 (Vazyme Biotech, New Jersey, USA) and TruePrepTM Index Kit V2 (Vazyme Biotech, New Jersey, USA) and was examined by Agilent High Sensitivity DNA Kit (Agilent Technologies, Inc, California, USA). Finally, the sequencing library was quantified by Illumina DNA Standards and Primer Premix Kit (KAPA Biosystems, Boston, USA), and massively parallel sequenced on Illumina HiSeq 2500 system.

### Statistical Analysis

Statistical analysis was performed using SPSS17.0 version. The difference of categorical data was compared using Chi-square test. The difference of measurement data was compared by analysis of variance. *p* < 0.05 was considered to be statistical significance.

## Results

A total of 401,660 newborns were screened by expanded newborn screening program (**Figure 1**). After initial screening, 11,247 (2.80%) newborns, who had positive results, were recalled for a new specimen. However, only 10,606 (94.30%) newborns with an initial positive result were collected a new specimen. After a repeated test, 732 (6.90%) newborns with a second positive result were determined to be suspect positive, and 720 (98.36%) of them were referred to diagnostic testing. Finally, 153 infants were diagnosed with one of IMEs and treated, and 138 of them were referred to genetic analysis. As SCADD and 3-MCCD were diseases with questionable phenotype, the overall incidence (excluding SCADD and 3-MCCD) was 1/3163. The comparison of all characteristics between normal newborns and patients did not reach at significant difference, including age at testing (p = 0.574), gender (p = 0.260), gestational age (p = 0.691), birth weight (p = 0.795), number of fetus (p = 0.988), register region (p = 0.571), and household registration (p = 0.166). The characteristics of newborns screened by expanded newborn screening program were shown in **Table 2**.

**Table 2:** Characteristics of newborns screened by expanded newborn screening program.

	Newborns without targeted IMEsN = 401,507	PatientsN = 153	*p*
Age at initial testing (days, mean ± SD)	7.35 ± 8.14	6.98 ± 6.08	0.574
Gender			
Male	210,273	86	0.260
Female	191,194	65	
No record	41	0	
Gestational age (weeks)			
<32	1,915	0	0.691
32∼36	18652	7	
>37	379861	146	
No record	1232	0	
Birth Weight (g)			
<1,500	607	0	0.795
1,500–1,999	1,986	0	
2000–2499	9,904	4	
>2,500	377,858	149	
No record	11,305	0	
Number of fetus			
Singleton	398,649	152	0.988
Twins	2,992	1	
Triplet	19	0	
Register region			
Suzhou	229,793	91	0.571
Others	171,867	62	
No record	0	0	
Household registration			
Urban	245,863	102	0.166
Rural	155,797	51	
No record	0	0	

**Figure 2:**
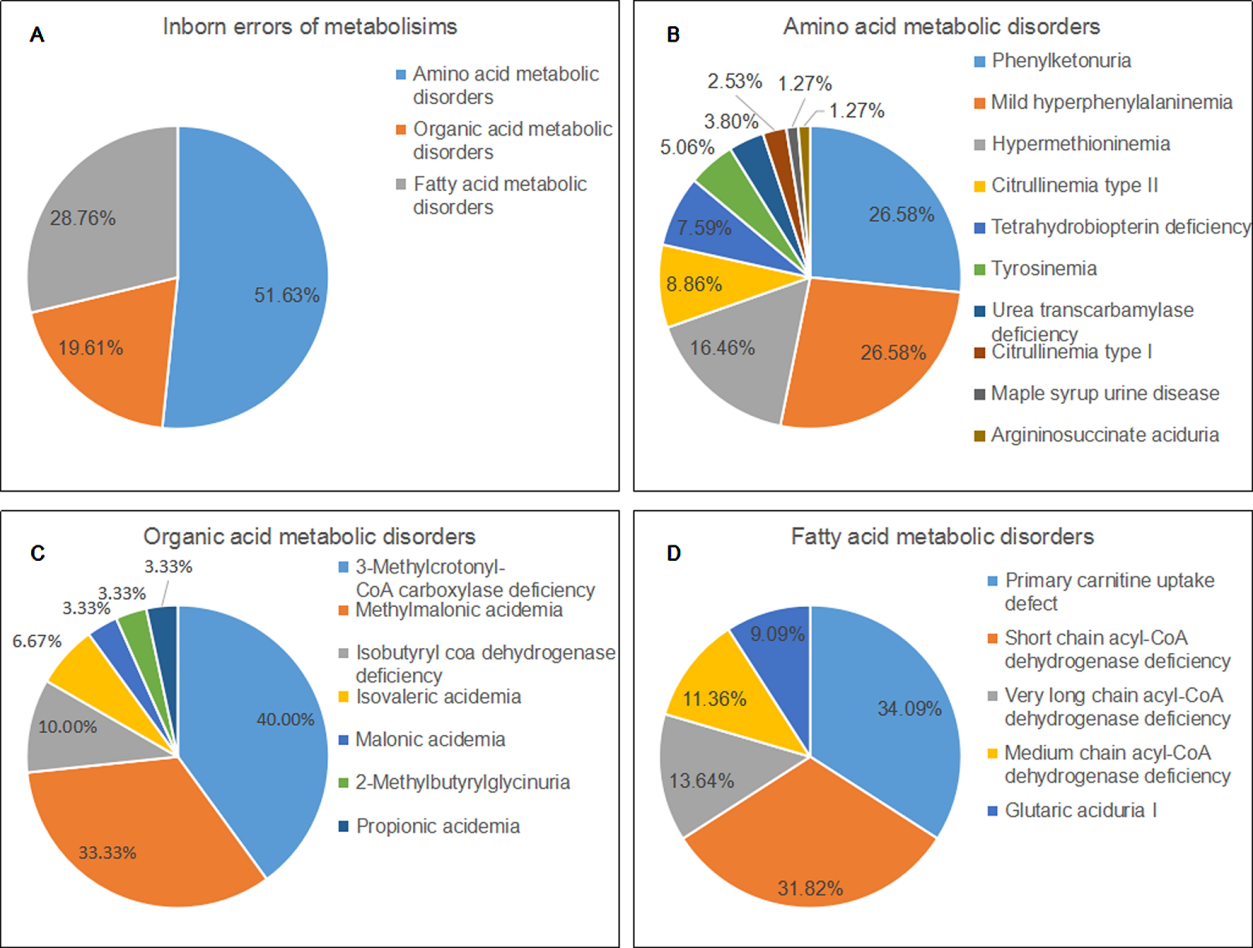
Disease spectrum and distribution of inborn errors of metabolisms. **(A)** The percentage of three categories of inborn errors of metabolisms. **(B)** the percentage of different kinds of amino acid metabolic disorders. **(C)** the percentage of different kinds of organic acid metabolic disorders. **(D)** the percentage of different kinds of fatty acid metabolic disorders.

Of 22 IEMs, 10 were amino acid metabolic disorders (AAMDs), 7 were organic acid metabolic disorders (OAMDs), and 5 were fatty acid metabolic disorders (FAMDs). The AAMDs were the most common diseases, accounting for 51.63% of patients, followed by FAMDs (19.61%) and OAMDs (28.76%). The overall prevalence of AAMDs, FAMDs, and OAMDs was 1/5,084, 1/11,814, and 1/10,041, respectively. HPA may be induced by PAH defect or tetrahydrobiopterin deficiency. A total of 48 infants with HPA were found, including 42 (87.5%) infants with PAH defect and 6 (12.5%) infants with tetrahydrobiopterin deficiency caused by PTPS (MIM* 612719) defect. The incidence of HPA, PAH defect, and PTPS defect were 1/8,368, 1/9,563, and 1/66,943, respectively. Furthermore, 42 infants with PAH defect were classified into two groups: 21 (50%) infants with PKU (≥360 μmol/L Phe) and 21 (50%) infants with M-HPA (120 μmol/L to 360 μmol/L Phe) (Chen et al., 2015). Of the 10 AAMDs, PKU and M-HPA were the most common diseases, accounting for 26.58% of patients, respectively, followed by hypermethioninemia (16.46%). The prevalence of single AAMD ranged from 1/401,660 to 1/19,127. Of the 7 OAMDs, 3-MCCD was the most common disease, accounting for 40.00% of patients, followed by methylmalonic acidemia (MMA; MIM# 251000) (33.33%). The prevalence of single OAMD ranged from 1/401,660 to 1/33,412. Of the 5 FAMDs, PCUD was the most common disease, accounting for 34.09% of patients, followed by SCADD (31.82%), VLCADD (13.64%), and MCADD (11.36%). The prevalence of single FAMD ranged from 1/100,411 to 1/26,777. All the above data were shown in **Figure 2** and **Table 3**.

**Table 3:** The spectrum and incidence of conditions from 401660 newborns screened by expanded newborn screening program.

Conditions	Patients	Estimated incidence	95%CI
Amino acid metabolic disorders	79	1/5,084	1/6,378–1/4,102
Phenylketonuria	21	1/19,128	1/30,093–1/12,729
Mild hyperphenylalaninemia	21	1/19,128	1/30,093–1/12,729
Hypermethioninemia	13	1/30893	1/55,556–1/18,532
Citrullinemia type II	7	1/57,372	1/131,199–1/29,011
Tetrahydrobiopterin deficiency	6	1/66,934	1/165,153-1/32,185
Tyrosinemia	4	1/100,411	1/316,056–1/41,632
Ornithine transcarbamylase deficiency	3	1/133,887	1/526,316–1/49,188
Citrullinemia type I	2	1/200,843	1/1,197,891–1/60,790
Maple syrup urine disease	1	1/401660	1/8,025,682–1/ 81,433
Argininosuccinate aciduria	1	1/401,660	1/8025682–1/81,433
Organic acid metabolic disorders	30	1/13,389	1/19,486–1/9,497
3-Methylcrotonyl-CoA carboxylase deficiency	12	1/33,412	1/61,767–1/19,689
Methylmalonic acidemia	10	1/40,166	1/79,051–1/22,533
Isobutyryl coa dehydrogenase deficiency	3	1/133,887	1/526,316–1/49,188
Isovaleric acidemia	2	1/200,843	1/1,197,891–1/60,790
Malonic acidemia	1	1/401,660	1/8,025,682–1/81,433
2-Methylbutyrylglycinuria	1	1/401,660	1/8,025,682–1/81,433
Propionic acidemia	1	1/401,660	1/8,025,682–1/81,433
Fatty acid metabolic disorders	44	1/9,129	1/11,669–1/6,863
Primary carnitine uptake defect	15	1/26,777	1/46,083–1/16,609
Short chain acyl-CoA dehydrogenase deficiency	14	1/28,690	1/50,403–1/17,516
Very long chain acyl-CoA dehydrogenase deficiency	6	1/66,934	1/165,153–1/32,185
Medium chain acyl-CoA dehydrogenase deficiency	5	1/80,332	1/219,250–1/36,245
Glutaric aciduria type I	4	1/100,411	1/316,056–1/41,632

With regard to genetic analysis, 89 reported mutations and 51 novel mutations were detected in 138 patients with one of IEMs (**Table 4**). All common IEMs affecting more than 10 patients had mutational hotspots. In 12 patients with PKU, 17 mutations were detected and the c.728G > A was the most common mutations in *PAH* gene, accounting for 20.8% of mutational alleles and 41.7% of patients, followed by c.611A > G (8.3% and 16.7%), c.721C > T (8.3% and 16.7%), and c.498C > G (8.3% and 8.3%). All the four hot mutations are pathogenic (www.ncbi.nlm.nih.gov/clinvar). In 18 patients with M-HPA, 19 mutations were detected and the c.158G > A was the most common mutations in *PAH* gene, accounting for 25.0% of mutational alleles and 50.0% of patients, followed by c.1238G > C (11.1% and 22.2%), c.728G > A (8.3% and 16.7%), and c.1315+6T > A (8.3% and 16.7%). However, the c.158G > A has a uncertain significance of pathogenicity (www.ncbi.nlm.nih.gov/clinvar). In 15 patients with PCUD, nine mutations were detected and the c.1400C > G was the most common mutation in *SLC22A5* (MIM* 603377) gene, accounting for 50.0% of mutational alleles and 80% of patients, followed by c.51C > G (13.3% and 26.7%) and c.760C > T (13.3% and 26.7%). All the three mutations are pathogenic and/or likely pathogenic (). In 14 patients with SCAD deficiency, nine mutations were detected and the c.1031A > G was the most common mutation in *ACADS* (MIM* 606885) gene, accounting for 42.9% of mutational alleles and 71.4% of patients, followed by c.164C > T (17.9% and 28.6%) and c.1130C > T (10.7% and 21.4%). The two mutation c.1031A > G and c.164C > T are likely pathogenic, and the c.1130C > T had conflicting interpretations of pathogenicity (www.ncbi.nlm.nih.gov/clinvar). Hypermethioninemia is able to be inherited by dominant transmission of *MAT1A* (MIM* 610550) gene. In 13 patients with hypermethioninemia, five mutations were detected and the c.791G > A was the most common mutation in *MAT1A* gene, accounting for 71.4% of mutational alleles and 76.9% of patients. The c.791G > A is pathogenic (www.ncbi.nlm.nih.gov/clinvar) and dominantly inherited. In 12 patients with 3-Methylcrotonyl-CoA carboxylase deficiency, 13 mutations and 2 mutations were detected in *MCCC1* (MIM* 609010) gene and *MCCC2* (MIM* 609014) gene. The most common mutation is c.639+2T > A of *MCCC1* gene, accounting for 16.7% of mutational alleles and 33.3% of patients, followed by c.863A > G of *MCCC1* gene (12.5% and 25.0%). The c.639+2T > A is pathogenic, but the c.863A > G has uncertain significance of pathogenicity (). Of 10 patients with MMA, 7 carried *MMUT* (MIM* 609058) gene mutations and 3 carried *MMACHC* (MIM* 609831) gene mutations. In all patients with MMA, the most common mutation was c.1663G > A of *MMUT* gene, accounting for 15.0% of mutational alleles and 30% of patients, followed by c.729_730insTT of *MMUT* gene (10.0% and 20.0%) and c.609G > A of *MMACHC* gene (10.0% and 20.0%).

**Table 4:** Mutations detected in patients with inborn error of metabolism identified by expanded newborn screening.

Conditions (OMIM number)	Gene (OMIM number)	Mutation alleles number	Nucleotide variant	Amino acid variant	Reported	Pathogenic	RF%	Cases	Accounting for patients (%)
Phenylketonuria (261,600)	*PAH* (612,349)	24						12	
		5	c.728G > A	p.R243Q	Y	P	20.8	5	41.7
		2	c.611A > G	p.Y204C	Y	P/LP	8.3	2	16.7
		2	c.721C > T	P.R241C	Y	P	8.3	2	16.7
		2	c.498C > G	p.Y166X	Y	P	8.3	1	8.3
		1	c.125A > T	p.K42I	Y	NP	4.2	1	8.3
		1	c.158G > A	p.P53H	Y	US	4.2	1	8.3
		1	c.208_210delTCT	p.S70del	Y	P	4.2	1	8.3
		1	c.331C > T	p.R111T	Y	P	4.2	1	8.3
		1	c.442-1G > A	/	Y	P	4.2	1	8.3
		1	c.722delG	R241Pfs	Y	LP	4.2	1	8.3
		1	c.722G > A	p.R241H	Y	P	4.2	1	8.3
		1	c.740G > T	p.G247V	Y	P/LP	4.2	1	8.3
		1	c.827T > G	p.M276R	Y	NP	4.2	1	8.3
		1	c.929C > T	p.S310F	Y	P	4.2	1	8.3
		1	c.1223G > A	p.R408Q	Y	P	4.2	1	8.3
		1	c.1238G > C	p.R413P	Y	P	4.2	1	8.3
		1	c.1264G > A	p.E422K	Y	NP	4.2	1	8.3
Mild hyperphenylalaninemia (261,600)	*PAH* (612,349)	36						18	
		9	c.158G > A	p.P53H	Y	US	25.0	9	50.0
		4	c.1238G > C	p.R413P	Y	P	11.1	4	22.2
		3	c.728G > A	p.R243Q	Y	P	8.3	3	16.7
		3	c.1315+6T > A	/	Y	LP	8.3	3	16.7
		2	c.1174T > A	p.F392I	Y	NP	5.6	2	11.1
		1	c.208_210delTCT	p.S70del	Y	P	2.8	1	5.6
		1	c.310G > T	p.A104S	N	US	2.8	1	5.6
		1	c.331C > T	p.R111X	Y	P	2.8	1	5.6
		1	c.464G > A	p.R155H	Y	P	2.8	1	5.6
		1	c.721C > T	p.R241C	Y	P	2.8	1	5.6
		1	c.722G > A	p.R241H	Y	P	2.8	1	5.6
		1	c.754C > T	p.R252W	Y	P	2.8	1	5.6
		1	c.770G > T	p.G257V	Y	LP	2.8	1	5.6
		1	c.782G > A	p.R261Q	Y	P	2.8	1	5.6
		1	c.977G > A	p.W326X	Y	P	2.8	1	5.6
		1	c.1301C > A	p.A434D	Y	LP	2.8	1	5.6
		1	c.1123C > G	p.Q375E	Y	NP	2.8	1	5.6
		1	1197A > T	p.V399X	Y	P	2.8	1	5.6
		1	c.1199G > A	p.R400K	Y	LP	2.8	1	5.6
Primary carnitine uptake defect (212,140)	*SLC22A5* (603,377)	30						15	
		15	c.1400C > G	p.S467C	Y	P/LP	50.0	12	80.0
		4	c.51C > G	p.F17L	Y	LP	13.3	4	26.7
		4	c.760C > T	p.R254X	Y	P	13.3	4	26.7
		2	c.497+1G > T	/	N	US	6.7	2	13.3
		1	c.394-1G > T	/	Y	LP	3.3	1	6.7
		1	c.428C > T	p.P143L	N	US	3.3	1	6.7
		1	c.652+1G > A	/	Y	P	3.3	1	6.7
		1	c.1252C > T	p.Q418X	Y	P	3.3	1	6.7
		1	c.1462C > T	p.R488C	Y	US	3.3	1	6.7
Short chain acyl-CoA dehydrogenase deficiency (201,470)	ACADS (606,885)	28						14	
		12	c.1031A > G	p.E344G	Y	LP	42.9	10	71.4
		5	c.164C > T	p.P55L	Y	LP	17.9	4	28.6
		3	c.1130C > T	P377L	Y	CIP	10.7	3	21.4
		2	c.322G > A	p.G108S	Y	LP	7.1	2	14.3
		2	c.737G > A	p.C246T	N	US	7.1	2	14.3
		1	c.77A > G	p.H26R	N	US	3.6	1	7.1
		1	c.973C > T	p.R325W	Y	CIP	3.6	1	7.1
		1	c.1054G > A	p.A352T	Y	US	3.6	1	7.1
		1	c.1055C > T	p.A352V	N	US	3.6	1	7.1
Hypermethioninemia (250,850)	MAT1A (610,550)	14*						13	
		10	c.791G > A	p.R264H	Y	P	71.4	10	76.9
		1	c.533C > T	p.P177L	N	US	7.1	1	7.7
		1	c.572_592dup		N	LP	7.1	1	7.7
		1	c.776G > T	p.A259V	Y	P	7.1	1	7.7
		1	c.790C > T	p.R264C	Y	P	7.1	1	7.7
3-Methylcrotonyl-CoA carboxylase deficiency (210,200 and 210,210)		24						12	
	MCCC1 (609,010)	4	c.639+2T > A	p.S164Rfs*3	Y	P	16.7	4	33.3
		3	c.863A > G	p.E288G	Y	US	12.5	3	25.0
		1	c.181G > T	p.A61S	N	US	4.2	1	8.4
		1	c.190G > A	p.V64M	N	US	4.2	1	8.4
		1	c.388G > A	p.G130S	Y	US	4.2	1	8.4
		1	c.416C > T	p.T139I	N	US	4.2	1	8.4
		1	c.490delA		N	US	4.2	1	8.4
		1	c.872C > T	p.A291V	Y	US	4.2	1	8.4
		1	c.1069G > T	p.E357X	N	LP	4.2	1	8.4
		1	c.1103delG	p.G368Vfs*70	N	LP	4.2	1	8.4
		1	c.1136G > A	p.G379D	N	US	4.2	1	8.4
		1	c.1381G > T	p.V461F	N	US	4.2	1	8.4
		1	c.1679dupA	p.N560Kfs*10	Y	P	4.2	1	8.4
	MCCC2( 609,014)	2	c.577C > T	p.R193C	N	US	8.3	2	16.7
		1	c.592C > T	p.Q198X	N	LP	4.2	1	8.4
		3	undetectable	–	–	–	12.5	3	25.0
Methylmalonic acidemia (251,000)		20						10	
	MMUT (609,058)	3	c.1663G > A	p.A555T	Y	LP	15.0	3	30.0
		2	c.729_730insTT	p.D244Lfs	Y	P	10.0	2	20.0
		1	c.322C > T	p.R108C	Y	P	5.0	1	10.0
		1	c.454C > T	p.R152X	Y	P	5.0	1	10.0
		1	c.581C > T	p.P194L	N	US	5.0	1	10.0
		1	c.755dupAA	p.H252QfsX6	N	LP	5.0	1	10.0
		1	c.1280G > A	p.G427D	Y	P	5.0	1	10.0
		1	c.1677-1G > A	p.R559Sfs*14	Y	P	5.0	1	10.0
		1	c.2080C > T	p.R694W	Y	P	5.0	1	10.0
		1	c.2131G > T	p.E711X	Y	LP	5.0	1	10.0
	MMACHC (609,831)	2	c.609G > A	p.W203X	Y	P	10.0	2	20.0
		1	c.394C > T	p.R132X	Y	P	5.0	1	10.0
		1	c.567dupT	p.190Yfs*13	Y	P	5.0	1	10.0
		1	c.658_660del	p.L220del	Y	P	5.0	1	10.0
		2	undetectable	–	–	–	10.0	2	20.0
Citrullinemia (605814 and 603,471)		16						8	
	SLC25A13 (603,859)	4	c.IVS16ins3Kb	/	Y	P	25.0	4	50.0
		3	c.852_855delTATG	p.M285Pfs	Y	P	18.8	3	37.5
		1	c.851_854delGTAT	p.Met284fs	Y	LP	6.3	1	12.5
		1	c.1078C > T	p.R360X	Y	P	6.3	1	12.5
		1	c.1399C > T	p.R467X	N	LP	6.3	1	12.5
		4	undetectable	–	–	–	25.0	4	50.0
	ASS1 (603,470)	1	c.689G > C	p.G230A	Y	LP	6.3	1	12.5
		1	c.1004G > A	p.R355H	Y	US	6.3	1	12.5
Very long chain acyl-CoA dehydrogenase deficiency (609,016)	ACADVL (609,575)	13#						6	
		2	c.887_888delCT	p.P296Rfs*17	Y	P/LP	15.4	1	16.7
		2	c.1349G > A	p.R450H	Y	P/LP	15.4	2	33.3
		1	c.278-31_278-18del	/	Y	US	8.3	1	16.7
		1	c.553G > A	p.G185S	Y	P/LP	8.3	1	16.7
		1	c.642_643delCT	p.F214Lfs*38	N	LP	8.3	1	16.7
		1	c.838A > G	p.T280A	N	US	8.3	1	16.7
		1	c.878+34G > A	/	N	US	8.3	1	16.7
		1	c.895A > G	p.K299E	N	US	8.3	1	16.7
		1	c.1077G > A	p.A359A	N	US	8.3	1	16.7
		1	c.1280G > A	p.W427X	Y	LP	8.3	1	16.7
		1	c.1345G > C	p.E449Q	Y	US	8.3	1	16.7
Tetrahydrobiopterin deficiency (233,910, 261,640, 612,716, 264,070, and 261,630)	PTS (612719)	12						6	
		5	c.259C > T	p.P87S	Y	P	41.7	3	50.0
		3	c.166G > A	p.V56M	Y	LP	25.0	3	50.0
		1	c.155A > G	p.N52S	Y	P	8.3	1	16.7
		1	c.272A > G	p.K91R	N	US	8.3	1	16.7
		1	c.277C > A	p.L93M	N	US	8.3	1	16.7
		1	c.286G > A	p.D96N	Y	P	8.3	1	16.7
Medium chain acyl-CoA dehydrogenase deficiency (201,450)	ACADM (607,008)	10						5	
		2	c.449_452delCTGA	p.T150Rfs	Y	P/LP	20.0	2	40.0
		1	c.589A > G	p.K197E	N	US	10.0	1	20.0
		1	c.790G > T	p.G264C	N	US	10.0	1	20.0
		1	c.970G > A	p.A324T	N	US	10.0	1	20.0
		1	c.1171A > G	p.M391V	N	US	10.0	1	20.0
		1	c.1238G > A	p.R413H	Y	US	10.0	1	20.0
		1	c.1247T > C	p.I416T	Y	CIP	10.0	1	20.0
		1	c.1248T > G	p.I416M	N	US	10.0	1	20.0
		1	undetectable	–	–	–	10.0	1	20.0
Tyrosinemia (276,700, 276,600, 276,710)		8						4	
	FAH (603,859)	1	c.5C > T	p.T2M	N	US	12.5	1	25.0
		1	c.236G > A	p.G79E	N	US	12.5	1	25.0
		2	undetectable	–	–	–	25.0	2	50.0
	HPD (609,695)	1	c.784G > A	p.A262T	N	US	12.5	1	25.0
		1	c.916C > T	p.R306X	N	LP	12.5	1	25.0
	TAT (613,018)	1	c.1162G > A	p.A388T	N	US	12.5	1	25.0
		1	c.1210G > A	p.A404T	N	US	12.5	1	25.0
Glutaric aciduria I (231,670)	GCDH (608,801)	8						4	
		2	c.1064G > A	p.R355H	Y	P	25.0	2	50.0
		1	c.158C > G	p.P53R	N	US	12.5	1	25.0
		1	c.554G > A	p.G185E	N	US	12.5	1	25.0
		1	c.892G > A	p.A298T	Y	P/LP	12.5	1	25.0
		1	c.916G > A	p.E306K	N	US	12.5	1	25.0
		1	c.1186G > C	p.D396H	N	US	12.5	1	25.0
		1	c.1240G > A	p.E414K	Y	P	12.5	1	25.0
Isobutyryl coa dehydrogenase deficiency (611,283)	ACAD8 (604,773)	6						3	
		3	c.1000C > T	p.R344C	Y	P/LP	50.0	3	100.0
		2	c.286C > A	p.G96S	N	US	33.3	2	66.7
		1	c.568-3C > G	/	N	US	16.7	1	33.3
Isovaleric acidemia (243,500)	IVD (607,036)	4						2	
		1	c.241C > T	p.R81X	N	LP	25.0	1	50.0
		1	c.466-29A > G	/	N	US	25.0	1	50.0
		1	c.1216A > G	p.T406A	N	US	25.0	1	50.0
		1	undetectable	–	–	–	25.0	1	50.0
Argininosuccinate aciduria (207,900)	ASL (608,310)	2						1	
		2	c.331C > T	p.R111W	N	LP	100.0	1	100.0
Maple syrup urine disease (248,600)	DBT (248,610)	2						1	
		2	c.1132C > T	p.Q378X	N	LP	100.0	1	100.0
Ornithine transcarbamylase deficiency (311,250)	OTC (300,461)	2						1	
		2	c.829C > T	p.R277Y	Y	P	100.0	1	100.0
Malonic acidemia (248,360)	MLYCD	2						1	
		2	c.482T > C	p.L161P	Y	US	100.0	1	100.0
2-Methylbutyrylglycinuria (611,283)	ACADSB (600,301)	2						1	
		2	C.1165A > G	p.M389V	N	LP	100.0	1	100.0
Propionic acidemia (606,054)	PCCA (232,000)	2						1	
		1	c.229C > T	p.R77W	Y	LP	50.0	1	100.0
		1	c.2002G > A	p.G668R	Y	P/LP	50.0	1	100.0

CIP, conflicting interpretations of pathogenicity; LP, likely pathogenic; N, no; P, pathogenic; RF, relative frequency; US, uncertain significance; Y, yes.

*one case carry two mutational alleles.

#, one case carry three mutational alleles.

Other IEMs including citrullinemia (MIM# 605814 and 603471), Tetrahydrobiopterin deficiency (MIM# 233910, 261640, 612716, 264070, and 261630), and Isobutyryl coa dehydrogenase deficiency (IBD; MIM# 611283) were also observed to have mutational hotspots. Of eight patients with Citrullinemia, seven were confirmed CTLN2 caused by mutations in *SLC25A13* (MIM* 603859) gene, and only one cittrullinemia I (CTLN 1; MIM# 215700) caused by mutations in *ASS1* (MIM* 603470) gene. In all patients with citrullinemia, the most common mutation was c.IVS16ins3Kb of *SLC25A13* gene, accounting for 25.0% of mutational alleles and 50.0% of patients, followed by c.852_855delTATG of *SLC25A13* gene (18.8% and 37.5%). Both c.IVS16ins3Kb and c.852_855delTATG of *SLC25A13* gene are pathogenic for CTLN2 (www.ncbi.nlm.nih.gov/clinvar). In six patients with Tetrahydrobiopterin deficiency, six mutations in *PTS* (MIM* 612719) gene were detected and the c.259C > T was the most common mutation, accounting for 41.7% of mutational alleles and 50.0% of patients, followed by c.166G > A (25.0% and 50.0%). Furthermore, the c.259C > T is pathogenic and the c.166G > A is likely pathogenic (www.ncbi.nlm.nih.gov/clinvar), and the two mutations account for 83.3% of patients. Interestingly, all the three patients with Isobutyryl coa dehydrogenase deficiency were heterozygous for the c.1000C > T variant of *ACAD8* (MIM* 604773) gene, and two patients were heterozygous for the c.286C > A variant, which has not been reported. The c.1000C > T of the *ACAD8* gene was reported to be pathogenic and likely pathogenic in patients with IBD (www.ncbi.nlm.nih.gov/clinvar). Obviously, the two mutations are hotspots and main causes for IBD.

## Discussion

PKU is an autosomal recessive genetic AAMD caused by deficiency of phenylalanine hydroxylase (PAH) (Blau et al., 2010). They were the most common IMEs identified by expanded newborn screening program, and the incidence of both were about 1/20,000. Up to now, more than 800 *PAH* mutations have been identified in patients with deficiency of PAH (Zhang et al., 2018). Some hotspot mutations exist in *PAH* gene and vary in different populations. For example, the most common *PAH* mutation is c.1222C > T in American (Kaul et al., 1994), IVS10-11G > A in Iranian (Zamanfar et al., 2017; Esfahani and Vallian, 2018; Rastegar Moghadam et al., 2018) and Spanish (Bueno et al., 2013; Aldámiz-Echevarría et al., 2016), c.168+5G > C in western Iranian (Alibakhshi et al., 2014), c.1238G > C in Japanese (Okano et al., 2011; Dateki et al., 2016), c.728G > A in Chinese (Zhou et al., 2012; Li et al., 2015; Zhang et al, 2018), c.781C > T in Karachays (Gundorova et al., 2018), c.1068C > A and c.728G > A in south Korean (Lee et al., 2008), c.1162G > A in Brazilian (Vieira Neto et al., 2018), c.782G > A in Syrian (Murad et al., 2013), and c.1222C > T in Australian (Ho et al., 2014). These different hotspot mutations suggested different origins. Similar to other Chinese populations, in our cohort, the c.728G > A is the most common *PAH* mutation that account for 20.8% of mutational alleles and 41.7% of classical PKU patients. However, in patients with M-HPA, the c.158C > A is the most common mutation that account for 25.0% of mutational alleles and 50.0% of patients. In a Japanese population, the c.158C > A also exhibited a relative higher prevalence in patients with hyperphenylalaninemia compared with PKU (Dateki et al., 2016). It appears that PAH deficiency has a correlation between genotype and clinical phenotype, and the c.158C > A could be considered as a marker for differentiating hyperphenylalaninemia from classical PKU.

PCUD is the second common IME. It shows a large variation of prevalence in different populations. For example, the prevalence of PCUD is 1/297 in Faroese (Rasmussen et al., 2014), 1/120,000 in Australian (Wilcken et al., 2003), 1/40,000 in Japanese (Koizumi et al., 1999), and 1/20,000–70,000 in American (Magoulas and El-Hattab, 2012). According to the available data, the prevalence of PCUD ranges from 1/45,000 to 1/8,000 in different areas of China (Han et al., 2012; Sun et al., 2017; Zheng et al., 2017; Guo et al., 2018), while the incidence of PCUD is 1/26,777 in Suzhou population. PCUD is caused by deficiency of organic cation transporter 2 (OCTN2) that results from variants in *SLC22A5* gene. The symptomatic patients presented a variety of clinical symptoms, including muscle weakness, dilated cardiomyopathy, hepatomegaly, encephalopathy, sudden infant death, feeding difficulty, recurrent pneumonia, vomiting, abdominal pain, and diarrhea (Han et al., 2014). These symptoms might be caused by different genotypes of PCUD (Rose et al., 2012; Chen et al., 2013). To date, more than 110 *SLC22A5* mutations have been reported and hotspot mutations vary in different population (Han et al., 2014). For example, C.844T > C is always observed in Caucasian PCUD patients (Burwinkel et al., 1999; Vaz et al., 1999; Wang et al., 1999), and c.1400C > G is the hotspot mutation in Southeast Asian (Koizumi et al., 1999; Nezu et al., 1999; Tang et al., 1999). However, in California patients, no obvious hotspot mutation was observed in PCUD patients (Gallant et al., 2017). This could be explained by the fact that those patients are multi-ethnic. With regard to Chinese, the hotspot mutations are similar in different regions. Chen et al. found the most common mutations were c.760C > T (32.9%), c.1400C > G (21.1%), and c.51C > G (14.5%) in Taiwan PCD patients (Chen et al., 2013). Han et al. reported that c.760C > T is the most common mutation in patients with symptomatic, and c.51C > G in patients with asymptomatic in Shanghai (Han et al., 2014). Guo et al. observed that c.1400C > G were the most common mutation in five Jining PCUD patients (Guo et al., 2018). Sun et al. also noted that the c.1400C > G was the most common mutation in seven Nanjing PCUG patients (Sun et al., 2017). Tan et al. found the c.51C > G is the most common mutations in Liuzhou PCUD patients (Tan et al., 2017). In agreement with most studies, the c.1400C > G is the most common mutation, with a relative frequency of 50% and accounting for 80% of Suzhou PCUD patients.

SCADD is the third prevalent disease of IMEs and the most prevalent disease of fatty acid metabolic errors in Suzhou population. SCADD had a wide spectrum of symptoms, including hepatic dysfunction, bilateral optic atrophy, vomiting, dysmorphic facial features, feeding difficulties, metabolic acidosis, epilepsy, ketotic hypoglycemia, developmental delay, lethargy, seizures, dystonia, myopathy, and hypotonia (Kılıç et al., 2017; Nochi et al., 2017). However, almost all patients with SCADD identified by newborn screening present no symptom or significant health tissue (Jethva et al., 2008; Waisbren et al., 2008; Huang et al., 2016; Zheng et al., 2017). Therefore, SCADD was not included in expanded newborn screening panels in many newborn screening centers (Dietzen et al., 2009; Mak et al., 2013; Smon et al., 2018). The reported incidence of SCADD is 1/25,000∼1/45,000 worldwide (Zytkovicz et al., 2001; Loukas et al., 2010; Lim et al., 2014). In consistence with the above reports, the incidence of SCADD in Suzhou population is 1/28,690. SCADD is caused by the deficiency of SCAD that is encoded by *ACADS* gene. Until now, about 70 variants have been reported to be pathogenic or likely pathogenic in *ACADS* gene, including two common variants, c.511C > T and c.625G > A (Tonin et al., 2016; Nochi et al., 2017). Most patients with SCAD deficiency carry two mutation alleles of the two common variants, or harbor one of them in combination with a rare variant in *ACADS* gene (van Maldegem et al., 2010), and the hotspot in Ashkenazi Jewish patients is a pathogenic c.319C > T mutation (Tein et al., 2008). However, 71.4% of Suzhou patients with SCAD deficiency carried the pathogenic mutation c.1031A > G, similar to Zhejiang SCADD patients (Huang et al., 2016), but different from Jining SCADD patients (Guo et al., 2018).

Several conditions, including deficiency in cystathionine β-synthase activity, tyrosinemia type I, and liver disease, could result in abnormal elevation of serum methionine. In this study, hypermethioninemia specially refers to abnormal elevated methionine caused by the abolished or reduced activity of hepatic methionine adenosyltransferase (MAT) I/III that are encoded by *MATA1* gene. More than 37 mutations described previously range from truncating mutations with no residual enzyme activity to mild missense mutations (Mudd., 2011; Chien et al., 2015). The prevalence of MAT I/III deficiency was reported to range from 1/110,000 to 1/20,000 in different newborn populations (Chien et al., 2005; Couce et al., 2008; Martins et al., 2012; Couce et al., 2013; Nagao et al., 2013). However, in mainland of China, the incidence is unreported. Our study reported a prevalence of 1/30,893 in Suzhou population of newborns. Of 13 Suzhou hypermethioninemia patients, 10 cases carried the dominant mutation c.791G > A (Pérez Mato et al., 2001; Muriello et al., 2017). Previous studies reported that the c.791G > A was the most prevalent mutation in Asian populations, such as Japanese, Chinese in Taiwan, and so on (Chien et al., 2005; Nagao et al., 2013). MATA1 deficiency is inherited either as autosomal-recessive or autosomal-dominant. Most *MAT1A* mutations give rise to autosomal recessive phenotypes, but several autosomal dominant mutations have also been observed, including c.776C > T, c.791G > A (Muriello et al., 2017), c.746G > A, and c.838G > A (Kim et al., 2016). With the exception of a few individuals with hypermethioninemia who present with abnormal neurological symptoms, most patients generally are free of major clinical manifestation. Hypermethioninemia shows clinical symptoms correlated to genotypes (Chou, 2000), while the c.791G > A could lead to mild hypermethioninemia. Of all *MATA1* mutations related to hypermethioninemia, the c.791G > A was the most common mutation identified in patients screened by expanded newborn screening (Couce et al., 2008; Martins et al., 2012; Couce et al., 2013). The c.791G > A mutation was the most prevalent mutation in Asian populations, including Japanese (Nagao et al., 2013) and Taiwan population (Chien et al., 2005). As expected, the c.791G > A was the most prevalent (80%) mutation in Suzhou newborns. In addition, another autosomal dominant mutation c.776G > T was found in one patient, and t one patient carried a novel heterozygous c.533C > T mutation. It appears that *MATA1* deficiency is mainly inherited *via* autosomal dominant mode in Suzhou population. Furthermore, we found no Suzhou patients exhibit obvious clinical abnormality. There is a wide range of clinical manifestations in individuals with mutations in *MAT1A* gene, from completely asymptomatic to neurological problems associated with brain demyelination (Furujo et al., 2012). As a result, we speculated that the extent of clinical manifestations is associated with the inherited mode, which needs further research.

3-MCCD is an autosomal recessive inborn error of leucine metabolism, resulting in leukodystrophy, developmental delays, hypoglycemia, acidosis, failure to thrive, lactic acidosis, and hyperammonemia (Elpeleg et al., 1992; de Kremer et al., 2002; Forsyth et al., 2016). Despite cases with 3-MCCD identified by expanded newborn screening are more than previous expected, a growing number of reports have shown that the majority of cases are in fact asymptomatic (Stadler et al., 2006; Arnold et al., 2008; Arnold et al., 2012; Lam et al., 2013; Ye et al., 2014; Rips et al., 2016). This suggests this condition might represent a biochemical phenotype, but not a disease, and therefore should be excluded from newborn screening panels (Wilcken, 2008; Forsyth et al., 2016; Rips et al., 2016). The 3-MCCD is classified into type I (MIM# 210200) and type II (MIM# 210210), caused by *MCCC1* gene and *MCCC2* gene, respectively. Until now, at least 66 *MCCC1* and 83 *MCCC2* mutations have been reported (Yang et al., 2015). The 3-MCCD was the most prevalent organic acid metabolic error and showed a large variation of incidence from 1/27,000 to 1/110,000 in different countries (Yang et al., 2015; Fonseca et al., 2016). Similar to another Chinese population (Yang et al., 2015), the incidence of 3-MCCD is about 1/33,412 in Suzhou population. Some previous reports revealed that *MCCC2* mutations were the main etiology of 3-MCCD (Uematsu et al., 2007; Cho et al., 2012; Grünert et al., 2012; Fonseca et al., 2016). However, in Zhejiang population, almost all (5/6) 3-MCCD patients carried one or two *MCCC1* mutations (Yang et al., 2015). Similarly, of 12 Suzhou patients with 3-MCCD, 75% (9/12) were caused by *MCCC1* mutations, which suggested the *MCCC1* mutations might be prevalent in China (Yang et al., 2015). It is worth mentioning that further genetic testing for more Chinese patients should be conducted to confirm the above conclusion. Several mutations were observed to have a relative high frequency in 3-MCCD patients, including c.838G > T (4/12), c.295G > A (3/56), c.1574+1G > A (3/56) in *MCCC2* gene and c.1155A > C (4/56) in *MCCC1* gene (Dantas et al., 2005; Cho et al., 2012). However, most studies did not observe mutational hotspot of the two genes (Stadler et al., 2006; Yang et al., 2015; Smon et al., 2018). Contrary to a previous report on Zhejiang patients, a mutational hotspot c.639+2T > A, that was observed in only one Zhejiang patient and predicted to be pathogenic (Yang et al., 2015), had a high prevalence (4/12) in Suzhou patients. This inconsistency could be caused by the diversity of races or a small sample size of patients. As a result, the mutational hotspot should be confirmed by further research based on a large number of patients.

MMA is a family of lethal, severe, and multisystems organic acid metabolic errors, which has a wide clinical spectrum, including anorexia, failure to thrive, hypotonia, developmental delay, progressive renal failure, functional immune impairment, optic nerve atrophy, and hematologic abnormalities. MMA is classified into two main forms according to phenotype, including isolated methylmalonic acidurias and combined methylmalonic aciduria and homocystinuria, and caused by the defects of 10 genes, including *MUT*, *MMAA*, *MMAB*, *CD320*, *MMADHC*, *LMBRD1*, *HCFC1*, *ABCD4*, *MCEE*, and *SUCLA2*. According to previous reports, the incidence of MMA was 1/50,000 in Japan (Shigematsu et al., 2002), 1/85,000 in Taiwan of China (Cheng et al., 2010), and 1/250,000 in Germany (Schulze et al., 2003). However, in mainland China, the incidence of MMA ranged from 1/3,920 to 1/26,000 (Tu, 2011; Han et al., 2016; Yin, 2016; Zhao et al., 2016). In Suzhou, the incidence of MMA is about 1/40,000, obviously higher than that in the above countries, but lower than that in Shandong, Henan, Beijing, Shanghai, and Taiwan. Recently, a study with large sample size, containing 1,003 MMA patients derived from 26 provinces or cities of China, demonstrated that MMA cblC and MMA mut were the two major types in China. Similar to the report by Liu et al., the MMA cblC and the MMA mut were also the most prevalent types in Suzhou. However, contrary to Liu et al. report, in Suzhou population, the MMA mut (six patients) was more prevalent than MMA cblC (three patients). Several hotspot mutations were reported in *MUT* gene and *MMADHC* gene. Han et al. reported that the c.729_730insTT of the *MMUT* gene was the most common mutation in Shanghai patients (Han et al., 2015). Liu et al. reported that the c.609G > A and the c.658_660delAAG of the *MMACHC* gene were the most common mutations in 70 unrelated MMA cblC patients (Liu et al., 2010). Yu et al. reported that the c.609G > A and the c.658_660delAAG were the most common mutations detected in 13 (81%) out of 16 MMA cblC patients (Yu et al., 2015). However, in Suzhou patients with MMA, the mutations c.1663G > A and c.729_730insTT of the *MMUT* gene and c.609G > A of the *MMACHC* gene were the most common mutations. Therefore, the hotspot mutations in Chinese patients with MMA might be c.609G > A and c.658_660delAAG of the *MMACHC* gene and might be c.1663G > A and c.729_730insTT of the *MMUT* gene.

Citrullinemia is an autosomal recessive disorder and a urea cycle disease leading to a wide spectrum of phenotypes, from life-threatening neonatal hyperammonemia to adult onset with mild symptoms, and even no manifestation (Saheki and Kobayashi, 2002; Gao et al., 2003; Häberle et al., 2003; Enns et al., 2005; Dimmock et al., 2008; Komatsu et al., 2008; Häberle et al., 2009; Salek et al., 2010). This disease is classified into CTLN 1 and CTLN 2, caused by mutations of *ASS1* gene and *SLC25A13* gene, respectively. The estimated prevalence of CTLN 1 and CTLN 2 is 1 in 44,300–200,000 (Kasper et al., 2010; Niu et al., 2010) and 1 in 7,100–230,000 (Yamaguchi et al., 2002; Kobayashi et al., 2003; Lu et al., 2005; Tabata et al., 2008; Kikuchi et al., 2012) based on expanded newborn screening, respectively. However, most CTLN2 cases were identified in countries of East Asia, especially in Japan. More than 137 mutations in ASS1 gene have been identified in worldwide patients (Diez-Fernandez et al., 2017). The c.1168G > A mutation is the most common mutation in several ethnic groups, including Germans, Spaniards, and Turks, but rare in Asians (Gao et al., 2003; Engel et al., 2009; Diez-Fernandez et al., 2017). Whereas, the c.421-2A > G is the most frequent mutation in East Asians (Kobayashi et al., 1995; Lee et al., 2013; Woo et al., 2013). However, in this study, only one CTLN 1 patient and two mutations of the *ASS1* gene were identified in Suzhou citurillinemia patients, which could be caused by ethnic specificity. With regard to CTLN2, a higher prevalence is observed in Suzhou patients compared to CTLN1. In previous studies, mutation detection of the *SLC25A13* gene was very high, greater than 90% of CTLN2 in East Asians (Yasuda et al., 2000; Saheki and Kobayashi, 2002; Yamaguchi et al., 2002; Kobayashi et al., 2003; Saheki et al., 2004; Lu et al., 2005). In contrast to CTLN1, the CTLN2 had a narrow spectrum of mutations in *SLC25A13* gene and highly clustered mutations (Woo et al., 2014). The c.851_854del mutation in *SLC25A13*, which was suspected to have a founder effect, was identified in CTLN2 patients throughout East Asian countries, such as China, Japan, Korea, and so on (Kobayashi et al., 1999; Tanaka et al., 2002; Imamura et al., 2003; Tabata et al., 2008). According to targeted mutation analysis, the frequency of overall mutations in CTLN2 is estimated to be 1/65–79 in Chinese, 1/69–73 in Japanese, 1/50–112 in Korean, and 1/70–97 in Taiwanese (Yamaguchi et al., 2002; Saheki and Kobayashi, 2002; Kobayashi et al., 2003; Saheki et al., 2004; Lu et al., 2005). In above countries, c.1177+1G>A and c.851_854del are the most common mutations in CTLN 2 patients. In Japanese patients, the c.1177+1G>A mutation (up to 43.1% of detected alleles) had the highest frequency, followed by c.851_854del mutation (up to 38.9%) (Saheki and Kobayashi, 2002; Yamaguchi et al., 2002). In Korea patients, the IVS16ins3kb and the c.851_854del were the most common mutations and were found at very high frequencies (100%) (Ko et al., 2007). In Hong Kong CTLN2 patients, the c.851_854del GTAT, IVS16ins3kb and c.852_855delTATG were the most common mutations of *SLC25A13* gene (Hui et al., 2014; Chong et al., 2018). In another Chinese population, the c.851_854del GTAT was the most common mutation and was observed in 100% CTLN2 patients (Lin et al., 2017). Some other studies also observed c.851_854del GTAT was the most common mutation in Chinese populations (Xing et al., 2010; Fu et al., 2011; Song et al., 2011). In our study, we found the c.852_855delTATG and the IVS16ins3kb were the most common mutations and accounted for 75.0% of cases. In addition, another reported hotspot mutation c.851_854del GTAT was also observed in one Suzhou patient. As a result, c.851_854del, c.852_855delTATG, and IVS16ins3kb might be the most common mutations and should draw more attention in genetic analysis of Chinese CTLN2 patients.

Tetrahydrobiopterin deficiency (or BH4 deficiency) is a rare inborn metabolic disorder characterized by the deficiency of tetrahydrobiopterin or BH4 and caused by mutations in one of the four genes, including *GCH1*, *PCBD1*, *PTS*, and *QDPR*. This condition is inherited by autosomal recessive pattern and has a wide spectrum of symptoms, including intellectual disability, progressive problems with development, movement disorders, difficulty swallowing, seizures, behavioral problems, and inability to control body temperature. The total prevalence of this condition is estimated 1/500,000 to 1/1,000,000 worldwide and was relative high in Asian populations. In mainland of China, BH4 deficiency accounted for 8.55% of patients with HPA (Ye et al., 2009), significantly higher than 1%–3% of HPA worldwide (Blau et al., 1996). In our study, the prevalence of BH4 deficiency in Suzhou newborns was about 1/67,000, higher than 1/140,000 in the mainland of China (Ye et al., 2009; Li et al., 2018). Two teams reported the mutations in *PTS* gene were the main cause of BH4 deficiency, accounting for more than 95% of Chinese patients (Ye et al., 2009; Ye et al., 2013; Li et al., 2018). Similar to the above reports, all these six Suzhou patients with BH4 deficiency were caused by mutations in *PTS* gene. To date, more than 90 mutations in *PTS* gene have been reported in different populations. There are several mutational hotspots in different regions. Wang and coworkers investigated 204 PTPS deficiency patients and found the c.259C > T (38.2%) in *PTS* gene was the most common mutation, followed by c.84-291A > G (11%) (Wang et al., 2018). Ye and coworkers investigated 136 Chinese patients with PTPS deficiency and found c.259C > T (42.9%) in *PTS* gene was the most common mutation, followed by c.286G > A (13.4%) (Ye et al., 2013). Similar to the report by Ye et al., Li and coworkers reported that the c.259C > T (31.82%) in *PTS* gene was the most common mutation, followed by c.286G > A (13.64%) (Li et al., 2018). In Suzhou BH4 deficiency patients, we also found the c.259C > T was the most common mutation, accounting for 41.7% of mutation alleles. However, the second common mutation was c.166G > A, accounting for 25% of mutation alleles. The two most common mutations accounted for 83.3% of BH4-deficiency patients. The remaining four mutation alleles were c.155A > G, c.272A > G, c.277C > A, and c.286G > A, respectively, and all of these mutations were reported previously. However, c.155A > G, c.272A > G, and c.286G > A were reported as common mutations in Chinese patients with BH4 deficiency (Ye et al., 2013; Li et al., 2018; Wang et al., 2018). This difference could be caused by a small sample size and different populations. As a result, these mutational hotspots are potential candidates for genetic analysis of Chinese patients with BH4 deficiency.

IBD deficiency is a very rare disorder characterized by disrupting the breakdown of Val. This condition is an autosomal recessively inherited disease and caused by mutations in the *ACAD8* gene. To the best of our knowledge, only 27 patients with IBD deficiency were reported in literature, and 28 mutations in the *ACAD8* gene were detected in these patients (Yun et al., 2015; Santra et al., 2016; Lin et al., 2018). Most patients with IBD deficiency were asymptomatic in neonatal period, and a few had developed features such as dilated cardiomyopathy, hypotonia, developmental delay, and speech delay (Yun et al., 2015; Lin et al., 2018). In Suzhou population of newborns, three patients with IBD deficiency were identified from more than 400,000 newborns, and all patients remained asymptomatic during treatment and follow-up. Two reported common mutations were detected, including c.1000C > T and c.286C > A in the *ACAD8* gene. Recently, Lin and coworker reported six Chinese patients with IBD deficiency and found the c.286C > A (7/14) was the most common mutation (Lin et al., 2018), followed by c.1000C > T. However, we found that the c.1000C > T was the most common mutation accounting for 50.0% (3/6) of mutational alleles in Suzhou patients with IBD deficiency, followed by c.286C > A (33.3%). As a result, the two mutations c.286C > A and c.1000C > T in the *ACAD8* gene could be considered as mutational hotspots resulting in IBD deficiency in Chinese population. In addition, a novel heterozygous mutation c.568-3C > G was found in one patient with IBD deficiency. Our results characterized the mutational hotspots in the *ACAD8* gene in Chinese patients with IBD deficiency and broaden the mutational spectrum of the *ACAD8* gene.

There were five patients who were affected with Argininosuccinic aciduria (ASA; MIM# 207900), Maple syrup urine disease (MSUD; MIM# 248600), ornithine transcarbamylase deficiency (OTD; MIM# 311250), 2-methylbutyrylglycinuria (MBG; MIM# 248360), and Malonic acidemia (MA; MIM# 248360), respectively. Despite these five kinds of IEMs are extremely rare in Suzhou population, the cases affected with one of these IEMs are homozygous for one of the mutations, including c.331C > T in *ASL*, c.1132C > T in *DBT*, c.829C > T in *OTC*, 1165A > G in *ACADSB*, and c.482T > C in *MLYCD*. Therefore, these mutations might be hotspots causing the above five IEMs. In Suzhou patients, the remaining six IEMs were not observed to have mutational hotspots, including MCAD deficiency, VLCAD deficiency, glutaric acidemia type I (GA-I; MIM# 231670), tyrosinemia (MIM# 276700, 276600, and 276710), isovaleric acidemia (IVA; MIM# 243500), and propionic acidemia (PROP; MIM# 606054). In 32 mutation alleles of 16 patients with one of above six IEMs, 24 reported mutations and 6 novel mutations were detected. This might be caused by a small sample size and further research is needed.

In summary, we have detected a few mutational hotspots and some novel mutations that account for most Suzhou patients with IEMs identified by expanded newborn screening that might be pathogenic. These mutational hotspots could be potential candidates for gene screening and these novel mutations expanded the mutational spectrum of IEMs. Our findings could be of value for genetic counseling and genetic diagnosis of IEMs.

## Data Availability Statement

The datasets generated for this study can be found in Sequence Read Archive using the accession number PRJNA566217.

## Ethics Statement

The protocol was reviewed and approved by Ethic committee of the affiliated Suzhou hospital of Nanjing Medical University.

## Author Contributions

BW and JX conceived and designed the research, analyzed data, and wrote the manuscript. TW, JM, and BW conducted experiments and reviewed the manuscript. QW conducted experiments. HL analyzed data and reviewed the manuscript. QZ and AG took part in diagnosis and treatment of infants with IME. All authors read and approved the manuscript.

## Funding

This study was supported by grants from the Jiangsu Maternal and Children Health Care Research Project (F201603 and F201715), the Jiangsu Provincial Medical Innovation Team (CXTDB2017013), the Suzhou Clinical Medical Expert Team (SZYJTD201708), the Jiangsu Maternal and Children Health Care Key Discipline (FXK201748), the Suzhou Science and Technology Support Program (SYS201649), the Suzhou Key Medical Center (SZZX201505), and Suzhou Industry Technology Innovation Project (SYS201770).

## Conflict of Interest

The authors declare that the research was conducted in the absence of any commercial or financial relationships that could be construed as a potential conflict of interest.

## Abbreviations

ASA, Argininosuccinate aciduria; BH4 deficiency, Tetrahydrobiopterin deficiency; BKT, beta-Ketothiolase deficiency; CACT, Carnitine-acylcarnitine translocase deficiency; CPT-Ia, Carnitine palmitoyltransferase I deficiency; CPY-II, Carnitine palmitoyltransferase II deficiency; EMA, Ethylmalonic encephalopathy; GA-I, Glutaric aciduria type I; H-ARG, Arginemia; HCY, Homocystinuria; H-MET, Hypermethioninemia; HMG, 3-Hydroxy-3-methylglutaric aciduria; H-ORN, Hyperornithinemia; H-PRO, Hyperprolinemia; H-TYR, Tyrosinemia; IBG, Isobutyrylglycinuria; IVA, Isovaleric acidemia; LCHADD, long-chain L-3-Hydroxy acyl-CoA dehydrogenase deficiency; MADD, mutiple Acyl-coa dehydrogenases deficiency; MAL, Malonic acidemia; MCADD, Medium chain acyl-CoA dehydrogenase deficiency; MCD, multiple Carboxylase deficiency (MCD); M-HPA, mild Hyperphenylalaninemia; MSUD, Maple syrup urine disease; MUT, Methylmalonic acidemia; NKHG, Nonketotic hyperglycinemia; OTC, Ornithine transcarbamylase deficiency; PCUD, Primary carnitine uptake defect; PKU, Phenylketonuria; PROP, Propionic acidemia; SCADD, Short chain acyl-CoA dehydrogenase deficiency; TFP, Trifunctional protein deficiency; TYR-I, Tyrosinemia type I; VLCADD, very long chain acyl-CoA dehydrogenase deficiency; 2M3HBA, 2-Methyl-3-hydroxybutyric aciduria; 2MBG, 2-Methylbutyrylglycinuria; 3-MCC, 3-Methylcrotonyl-CoA carboxylase deficiency; 3MGA, 3-Methylglutaconic aciduria.
