# Supplementary material for: Expanded Newborn Screening for Inborn Errors of Metabolism by Tandem Mass Spectrometry in Suzhou, China: Disease Spectrum, Prevalence, Genetic Characteristics in a Chinese Population
Source: Front Genet. 2019 Oct 29;10:1052. doi: 10.3389/fgene.2019.01052 (PMC6828960; doi:10.3389/fgene.2019.01052)
Supplement: Supplementary file 1 [file Table_1.docx]

**SUPPLEMENTARY TABLE 1 Dried blood spot metabolites measured by expanded newborn screening program.**

| Amino acids (umol/L) | Acylcarnitines (umol/L) |
| --- | --- |
| Alanine (ALA) | Hexanoylcarnitine(C6) |
| Arginine(ARG) | Methylglutarylcarnitine(C6DC) |
| Citrulline(CIT) | Octanoylcarnitine(C8) |
| Glycine(GLY) | Octenoylcarnitine(C8:1) |
| Leucine+Isoleucine+Proline-OH (LEU+ILE+PRO-OH) | Decanoylcarnitine(C10) |
| Methionine(MET) | Decenoylcarnitine(C10:1) |
| Phenylalanine(PHE) | Dodecanoylcarnitine(C12) |
| Tyrosine(TYR) | Dodecenoylcarnitine(C12:1) |
| Valine(VAL) | Tetradecanoylcarnitine(C14) |
| Ornithine(ORN) | Tetradecenoylcarnitine(C14:1) |
| Proline(PRO) | Tetradecadienoylcarnitine(C14:2) |
| Acylcarnitines (umol/L) | 3-Hydroxytetradecanoylcarnitine(C14-OH) |
| Carnitine free(C0) | Palmitoylcarnitine(C16) |
| Acetylcarnitine(C2) | Palmitoleylcarnitine(C16:1) |
| Propionylcarnitine(C3) | 3-Hydroxypalmitoleylcarnitine(C16:1-OH) |
| Malonylcarnitine(C3DC) | 3-Hydroxypalmitoylcarnitine(C16-OH) |
| Butyrylcarnitine+Isobutyrylcarnitine(C4) | Stearoylcarnitine(C18) |
| Methylmalonylcarnitine(C4DC) | Oleoylcarnitine(C18:1) |
| Isovalerylcarnitine+Methylbutyrylcarnitine(C5) | 3-Hydroxyoleoylcarnitine(C18:1-OH) |
| Tiglylcarnitine(C5:1) | Linoleoylcarnitine(C18:2) |
| Glutarylcarnitine(C5DC) | 3-Hydroxystearoylcarnitine(C18-OH) |
| 3-Hydroxyisovalerylcarnitine(C5-OH) | Succinylacetone(SA) |
